# Developing a WHO African Region mOral Health Curriculum for Community Health Workers

**DOI:** 10.5334/aogh.4655

**Published:** 2025-08-06

**Authors:** Brittany Seymour, Donna Hackley, Miriam Muriithi, Danielle Burgess, Nikki Aflatooni, Dahee Chung, Nithya Ramesh

**Affiliations:** 1 Harvard School of Dental Medicine, USA; 2 Ministry of Health, Kenya

**Keywords:** mHealth, oral health, global oral health, community health worker training

## Abstract

*Background:* Training allied health professionals in oral health promotion and disease prevention, and integrated into noncommunicable disease (NCD) management, has been shown to improve access to essential oral health services. Oral diseases in the WHO African region are a significant public health problem, and trained dental professionals are scarce.

*Objectives:* The WHO African Regional Office (WHO AFRO) aims to create a novel tiered oral health workforce, beginning with community health worker (CHW) training on oral health, and utilizing combined in-person and virtual/digital learning through mobile technologies (mOral Health). Successful scale of the program will assist in improving the oral health knowledge, skills, and behaviors of CHWs in Africa, as part of their essential packages of basic services.

*Approach:* Guided by a logic model framework, our approach for developing the mOral Health curriculum was based on a proven six-step model for curriculum development in health professions education. Steps 1–3 describe our approach for developing the training program: Step 1: Problem Identification and General Needs Assessment; Step 2: Targeted Needs Assessment; and Step 3: Goals and Objectives.

*Results:* Step 4 describes the resulting curriculum and educational strategies. This is the WHO African region’s first competency-based CHW training program universally accessible to all member states. Step 5 (Implementation) and Step 6 (Evaluation and Revision) are planned for subsequent work at a future stage of this project.

*Conclusion:* The mOral Health curriculum for CHWs in the WHO African region leverages digital technologies as part of the WHO mHealth initiative and aligns with the WHO Global Strategy on Oral Health. This mOral Health curriculum can lay the groundwork for further development of an evidence-based, tiered oral health workforce in Africa and will integrate oral health services into the WHO AFRO agenda for the prevention, control, and management of NCDs across the region.

## Background

Oral diseases are a significant public health problem affecting an estimated 3.5 billion people globally [1]. Despite persistently being the most prevalent noncommunicable condition worldwide, oral diseases (e.g., dental caries, oral cancers, periodontal disease, and noma) were not a part of the global health agenda until recently. The United Nations (UN) Declarations on Noncommunicable Diseases (NCDs) [2] and Universal Health Coverage (UHC) [3] now explicitly include oral health. Additionally, the Lancet Commission on Oral Health advocates for policy change and a commitment to global oral health within medicine and the global health agenda [4]. Perhaps most significantly, on January 21, 2021, the World Health Organization (WHO) adopted its first ever resolution on oral health during its annual World Health Assembly [5]. The resolution identified oral health as integral to the WHO global strategy on healthy aging [6], the 2030 Agenda for Sustainable Development [7], the Prevention and Control of Noncommunicable Diseases [2], and the UN Declaration on Universal Health Coverage [3]. The WHO recognized the tremendous burden of oral diseases and their negative impact on quality of life, economic productivity, and overall health and well-being across the lifespan [5]. The subsequent WHO Global Strategy and Action Plan on Oral Health (2023–2030) is now set to inform oral health planning worldwide [8]. These significant milestones cemented oral health’s place in the global health and development agenda and are paving the way for reducing the tremendous burden of oral diseases and their risk factors shared with other NCDs.

**Figure 1:**
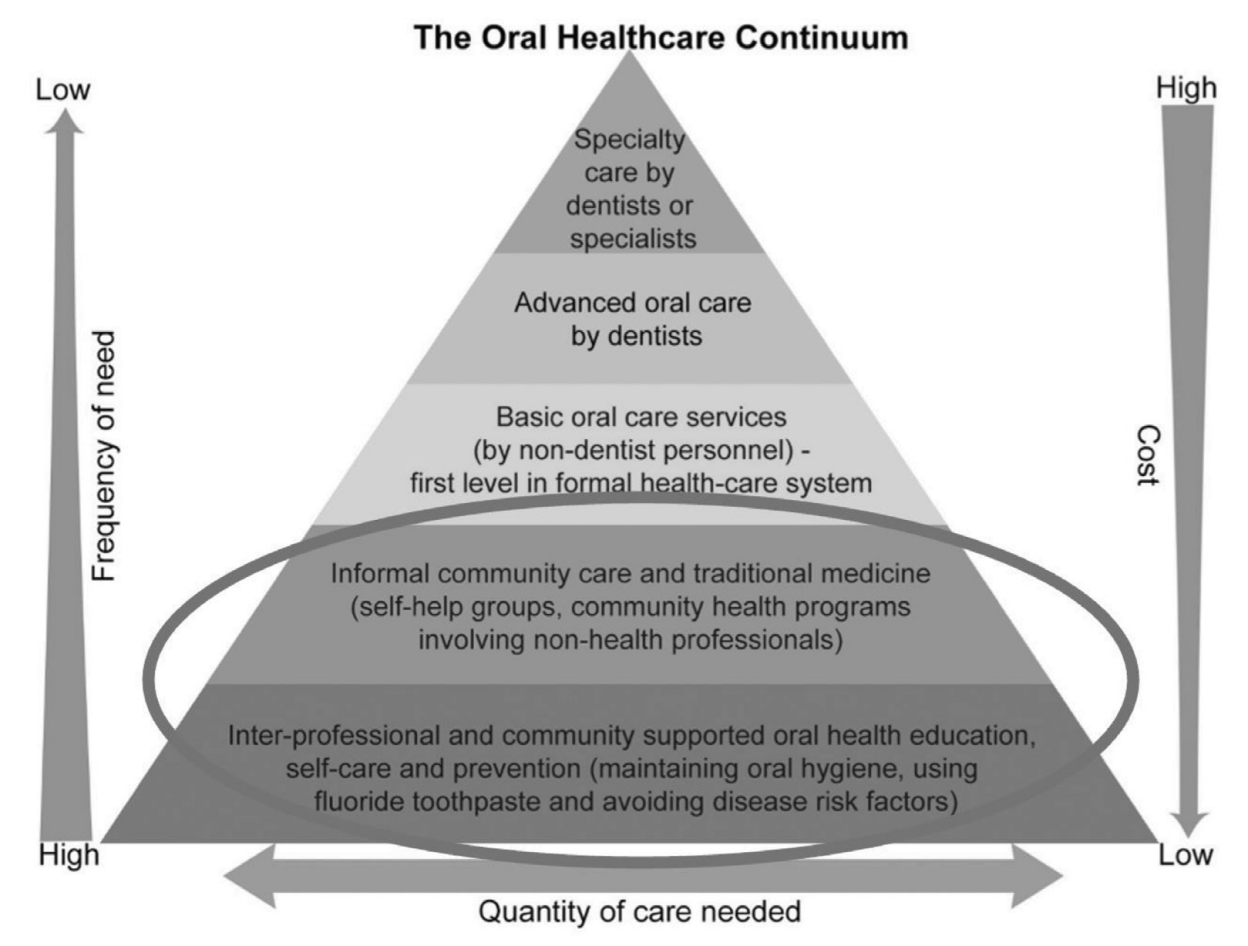
The Oral Health-Care Continuum, adapted and modified with permission from Hackley et al. [12] Original text and illustration copyright FDI World Dental Federation 2015. Maps, graphics, and original concept copyright Myriad Editions 2015 [13]. The focus of this project and the development of the mOral Health training for Community Health Workers are circled.

Oral diseases are contributing to the rising burden of NCDs in the WHO African Region (WHO AFRO), and it is estimated that NCD-related complications will be the most common cause of mortality in this region by the year 2030 [9]. Notably, the Africa Regional Summary of the WHO Global Oral Health Status Report found that the region experienced the sharpest increase in oral diseases compared to any other region globally in the last 30 years. The sparse availability of oral health professionals, largely concentrated in mostly urban areas, results in inequitable access to care services [10]. WHO AFRO region is approximately 1:23,000 [11] compared to the WHO recommended ratio of 1:7500 [12] and a US provider/population ratio of approximately 1:1600 [13]. Existing workforce models that require highly trained oral health professionals to deliver oral care at all levels of the healthcare system are not sufficient to manage the excessive burden of disease. The WHO Global Strategy on Human Resources for Health: Workforce 2030 aims to accelerate progress toward UHC and includes strategic workforce objectives to meet the needs of communities [14]. Existing evidence supports the need for a tiered oral health workforce that begins with disease prevention and health promotion by community-level workers, continues with providers in primary care settings, and then includes treatment delivery by highly skilled providers, including dentists and specialists. This tiered structure is known as the primary health continuum (Figure 1) [15, 16].

The WHO’s Global Strategy on Oral Health outlines strategic objectives for improving access to oral care through innovative workforce models that include competency-based training of nondentists and other allied health professionals [8]. In addition, the WHO Global Strategy on Human Resources for Health emphasizes the use of digital technologies and mobile devices to deliver e-training for workforce development as part of WHO’s global mHealth initiative [14, 17]. Thus, WHO AFRO aims to facilitate the creation of a tiered oral health workforce founded on community health worker (CHW) training in oral health promotion and disease prevention by encouraging the utilization of digital technologies and the integration of oral health into their basic packages of primary care services. CHWs are widely utilized in Africa and remain a vital component of primary care, essential for achieving UHC. However, to date there was no openly accessible, competency-focused oral health training program for CHWs in the African region. The aim of this project was to develop the first competency-based mOral Health curriculum for practicing CHWs in Africa to help meet the unmet need for oral health services in the region. The resulting curriculum aims to build the capacity of CHWs on oral health promotion and oral disease prevention and control, and to equip them with the knowledge and skills needed to triage and refer patients to health-care professionals who can provide adequate care. This paper describes the development of this novel training program and the curriculum that resulted. A subsequent manuscript will describe an implementation pilot exercise.

## Mapping Out the Approach: Needs Assessments and Identification of Specific Training Goals

**Figure 2:**
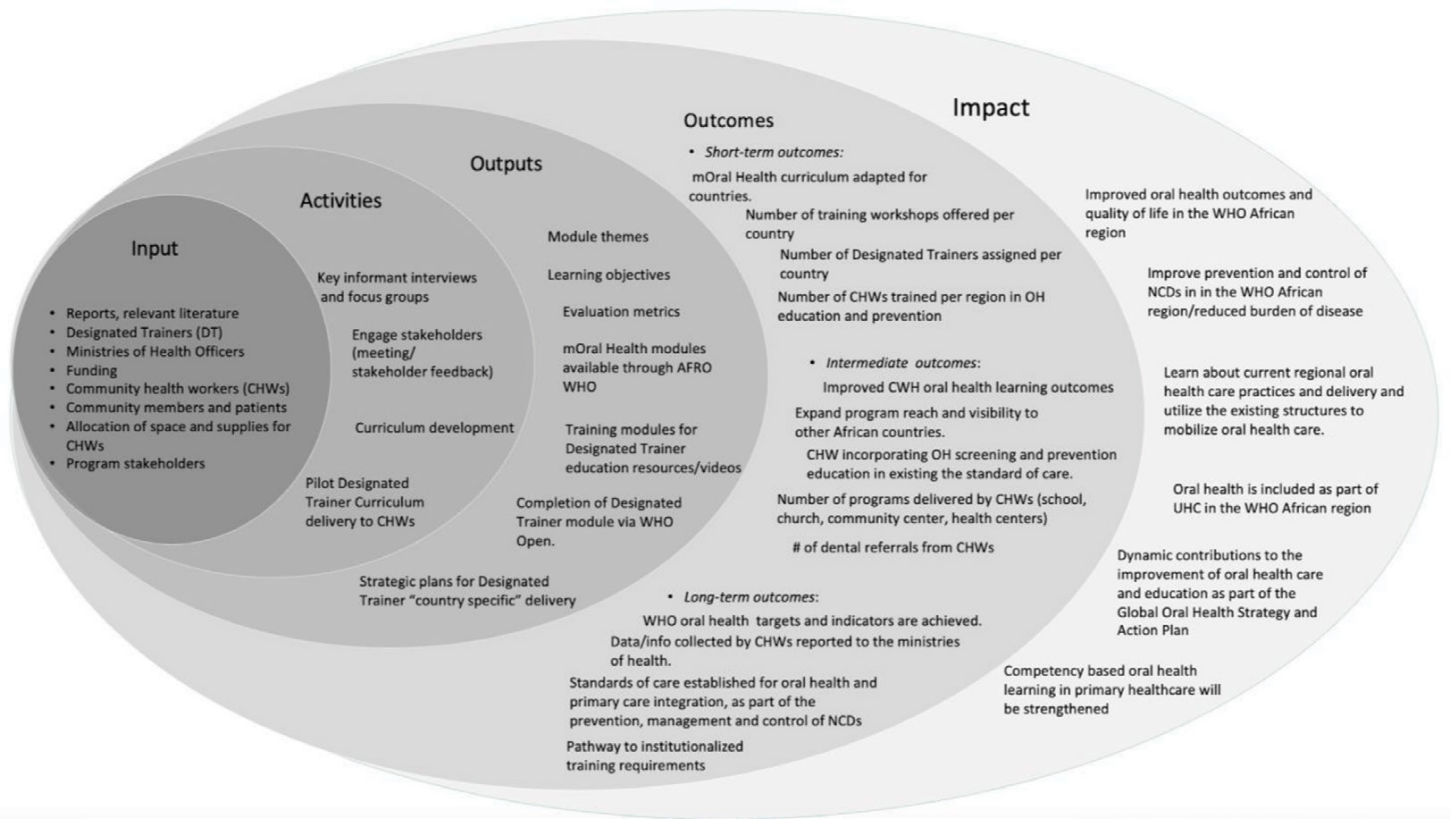
WHO AFRO Competency-Based mOral Health Curriculum Project Logic Model. DT: designated trainer; CHW: community health worker; OH: oral health; NCDs: noncommunicable diseases; WHO: World Health Organization; AFRO: Regional Office for Africa.

The logic model is a well-established organizational tool used to describe and present a systematic process for the design, implementation, data reporting, and evaluation of a program [18]. Creating a logic model was a substantial feature in the preliminary stages of developing this competency-based mOral Health curriculum for CHWs. The model illustrated the relationship between the input of available resources that inform and support the program’s key activities and the associated outputs, measurable outcomes, and intended overall long-term impact of the program. It also helped build consensus among stakeholders and identified checkpoints for measurable evaluation. Specifically, this model defined the sequence of intended outcomes in the short-, intermediate-, and long terms, allowing for a systematic and thorough approach for curriculum development, implementation, and monitoring and evaluation (Figure 2). This present logic model framework is dynamic and evolves to adapt to changing circumstances through regular updates, reviews, and revisions as the project progresses.

Ethical review was overseen by the Harvard Longwood Campus Institutional Review Board. The Institutional Review Board (IRB) of the Harvard Faculty of Medicine for protocol # IRB25-0474 determined that this submission is not human subject research as defined by Department of Health and Human Services (DHHS) regulations or Food and Drug Administration (FDA) regulations. The determination did not absolve the project team from complying with other research-related institutional and ethical requirements.

Guided by our logic model, our approach for developing the mOral Health curriculum for CHWs followed a proven six-step model for curriculum development in health professions education [19], which includes the following steps: Step 1: problem identification and general needs assessment; Step 2: targeted needs assessment; Step 3: goals and objectives; Step 4: educational strategies: Step 5: implementation; and Step 6: evaluation, feedback, and revision. This paper focuses on content creation, the approach for developing the training program in Steps 1–3, and describes the resulting curriculum in Step 4. Steps 5 and 6, which focus on implementation, are planned for subsequent work at a future stage of this project and are not reported here.

### Step 1: Problem identification and general needs assessment

Step 1 involved a general needs assessment to clarify the overall problem to be addressed. A major feature of the project development was iterative stakeholder feedback and a robust peer-review process with the intention to be diverse, representative, and inclusive. Four pilot countries were selected to support the early stages of this project, including curriculum design and development: Angola, Kenya, Liberia, and Senegal. The pilot countries were preselected by the WHO Regional Office for Africa prior to the start of this project, which aimed for representative diversity in language spoken, geographic region, economic level, and status of national oral health infrastructure. Pilot countries were asked to identify expert stakeholders who were knowledgeable and experienced individuals who would be impacted by and could impact the project. Stakeholders were invited to participate in all aspects of the design of the curriculum. Stakeholders included representatives from ministries of health and national governments, development partners and funding agencies, leaders in global health initiatives, representatives of regulatory bodies and professional organizations, health-care workers, community members, dental educators, and information technology professionals.

Building from policy milestones described previously, and to address potential challenges, obstacles, and questions broadly related to the challenge of developing a novel curriculum, we consulted multiple, evidence-based guidelines and reports that have been informed by systematic and collaborative processes. A literature review identified multiple WHO programs, declarations, and reports that were consulted throughout curriculum development. The WHO Promoting Oral Health in Africa report served as a primary guiding document in highlighting essential NCDs [20]. The WHO Guideline on Health Policy and System Support identifies the importance of CHWs in achieving UHC and provides evidence-based guidelines for CHW training programs [21]. The WHO Implementation Manual for Ending Childhood Dental Caries, a report of the WHO Prevention of Noncommunicable Diseases Oral Health Programme [22], emphasized the importance of CHWs in health care and serves as a second, oral health specific WHO report in alignment with the WHO oral health resolution [5] and FDI oral health continuum model [16].

### Step 2: Needs assessment for targeted learners (CHWs)

The general needs assessment conducted in collaboration with our stakeholders resulted in consensus to develop a competency-based mOral Health training for CHWs, aligning with initial project aims. Subsequently, a targeted needs assessment was conducted. First, we held four virtual focus group discussions, one with each of the pilot country stakeholder groups, to refine the overall project aims, synthesize our literature review for direct application to the project, and assess available competency frameworks for integrating oral health into CHW programs. Based on the outcomes from the focus groups, a project workbook was developed. The workbook was organized into four overarching sections: (1) curriculum themes/topic areas, (2) competencies and learning objectives, (3) the training and education levels most appropriate for CHWs, and (4) the environment in which learners are operating. Stakeholders filled the workbook with contextually relevant, experiential knowledge and curriculum recommendations.

Next, all stakeholders were invited to participate in the first of two half-day virtual Stakeholder Workshops. Stakeholders represented pilot countries’ Chief Dental Officers, Ministry of Health focal persons in the areas of NCDs, CHWs, country representatives from national medical and dental associations, country focal persons for policy and planning, funders, international NGOs, the WHO AFRO health workforce focal person, and the WHO EMRO oral health focal person. Workshop participants were presented with the results from the targeted needs assessments and focus group interviews and an overview of the proposed project. Stakeholders engaged in breakout discussions by language to actively discuss barriers and facilitators of project development and implementation. Participants utilized the workbook from the focus groups to build consensus on the objectives, scope, format, and content of this novel mOral Health curriculum for CHWs. Interpreters were present for all focus group sessions and the workshop.

### Step 3: Overall project goals, competencies, and objectives

Step 3 included defining specific goals for integrating oral health training into existing CHW programs, detailing competencies and measurable learning objectives, and outlining a strategy for the selection and preparation of designated trainers (DTs) to deliver the curriculum to CHWs and assess competency.

CHWs have been shown to be effective in community and social settings for addressing social determinants of health for the transformation of living conditions and community organization [21]. Stakeholders provided key recommendations, including (1) when selecting CHW trainees, education level should align with tasks to be performed; (2) membership in and acceptance of the community place of work is important; (3) formal certification should include recognition of training by relevant authorities following completion of preservice education requirements by designated, qualified trainers; (4) DTs are essential for conveying trainee competence in predetermined competency standards that are recognized by health authorities and national leadership; and (5) particular competency themes were determined most appropriate, including a focus on preventive services, integration into the wider health-care system, incorporation of the social determinants of health, and strengthening interpersonal skills and communication. Stakeholder recommendations offer each nation guidance on key aspects of the training while retaining flexibility to be customized for implementation.

Competency-based education focuses on the needs of the population and the desired learning outcomes necessary for trainees to meet that need. The desired outcome competencies are determined first, and the content is developed next, to support these predetermined outcomes in a standardized manner [23]. The 2015 Competency Matrix for Global Oral Health provided core global health competencies for community-level health workers [24]. WHO published a competency framework for UHC in 2022 [23]. These peer-reviewed, systematically developed competency frameworks served as a foundation for the development of more targeted measurable learning objectives and training topics for this mOral Health curriculum that could be integrated into existing trainings rather than serve as a siloed, stand-alone oral health curriculum.

Stakeholders recommended training that strikes a balance between theory and practice, including a combination of in-person and digital/virtual learning structures. Step 3 determined that ideal training modalities should include interprofessional training with emphasis on local priorities and supervised practical experiences. The WHO guidelines on optimizing CHW programs described trainee assessment and supervision as an important part of an overall strategy that becomes particularly relevant when implementing competency-based training. Supportive training supervision should include observation, assessment, mentorship, and feedback [21]. Competency-based formal certification should include recognition of training by relevant authorities upon completion of preservice skills evaluation by designated, qualified trainers. Thus, country-specific qualified DTs are essential for attesting to trainee competence in the predetermined competency standards [21]. This approach supported the rationale for ensuring that DTs are fully trained and qualified to mentor, support, and assess CHWs throughout their training. Stakeholders agreed that the curriculum should follow specific guidelines to be adaptable, easily understandable, and well supported, which were deemed an essential responsibility of DTs during delivery. Stakeholder feedback indicated that workforce personnel who currently train and supervise community-level workers should be able to adapt the curriculum for context, translate it to local languages if needed, and deliver content to CHWs with literacy levels varying from literate to illiterate. The DTs would likely derive from existing oral health personnel (when available), nurses, midwives, or community health assistants.

## Resulting Curriculum: Content, Teaching Strategies, and Student Evaluation

### Step 4: Educational strategy

#### Content development

Stakeholder feedback was again collected through the second of two stakeholder meetings and use of the collaborative workbook to reach consensus for the development of curricular content, pedagogy, and evaluation measures. A five-module structure aligned with the themes, competencies, and learning objectives that had emerged from Steps 1–3. Details of each module’s content, competencies, and learning objectives can be found in Table 1. Each module contains self-guided mHealth training, hosted on the open-access platform OpenWHO. While WHO AFRO does not directly administer the mOral health training, the module lives on OpenWHO, WHO’s interactive, web-based, knowledge-transfer platform offering open-access online courses to anyone anywhere [25]. OpenWHO grants a certificate upon successful completion of all online training. Implementing bodies, such as ministries of health, within each country elect to utilize the open-access module and accept responsibility for in-person trainings and evaluations for which they may choose to confer certificates of completion. In addition, while not entirely within the scope of this project, to support future implementation efforts, the course was designed to follow the WHO Community Health Workers Guidelines report [21]. This project’s curricular content is available in the form of both PowerPoint presentations and as narrated video presentations with scripted voice-overs with corresponding transcripts. PowerPoint presentations and videos are downloadable for optimal flexibility. Original graphics and images were created through an iterative peer-review process, engaging peers who either live locally in Africa or who have studied the region significantly, to help ensure they were contextually and regionally relevant. In collaboration with the Center for African Studies at Harvard University, narrators were identified from corresponding countries in the region and provided authentic narration to the scripted videos. All course materials were reviewed by our stakeholder team and underwent several rounds of edits accordingly.

**Table 1:** mOral Health curriculum by module, including module description, themes, supporting competencies (see also Appendices 1 and 2), learning objectives, and options for integration into existing trainings for community health workers.

WHO REGIONAL OFFICE FOR AFRICA MORAL HEALTH TRAINING FOR COMMUNITY HEALTH WORKERS			
**Module Content:** PowerPoint presentation, corresponding narrated videos, video transcripts, formative assessment (multiple-choice quiz), references; available in English, French, and Portuguese			
**Module**	**Competencies**	**Learning Objectives**	**Integration Options**
**Module 1: Introduction to oral health** Overview of oral health’s relationship and importance to overall health and well-being across the lifespan Basic oral health foundations (anatomy and physiology)	UHC: 1, 3–6, 10, 18, 19, 21–24 GOH: 1.1.4.,1.2.3., 2.1.2., 3.1.1., 3.2.1.	LO 1.1 Explain, using examples, the relationship between oral health and overall health and well-being across the life course LO 1.2 Describe healthy individual behaviors and community factors that promote overall and oral health LO 1.3 Recognize the healthy condition of infant, child, adolescent, and adult teeth and soft tissues	LO 1.1 Within existing training modules on healthy human anatomy and physiology LO 1.2 Within existing training on the social determinants of health LO 1.3 Within existing training modules on healthy human anatomy and physiology
**Module 2: Introduction to oral diseases and conditions** Overview of the relationship between oral diseases and conditions and other common noncommunicable diseases Common oral illnesses and conditions (caries, gingivitis, periodontal disease, oral cancers)	UHC: 1, 3–6, 10, 18, 19, 21–24 GOH: 1.1.2., 1.1.3., 1.2.1., 1.2.2.	LO 2.1 Explain common risk factors for oral and systemic illness/NCDs such as tobacco, alcohol, and sugar LO 2.2 Outline basic disease processes of dental caries, gingivitis, periodontal disease, and oral cancers LO 2.3 Screen for and recognize signs and symptoms of common oral diseases, including dental caries, gingivitis, periodontal disease, oral cancers, noma, and infant oral mutilation (and/or other locally relevant harmful oral health practices)	LO 2.1 Within NCD training; within maternal and child health training LO 2.2 Within existing training on disease processes; within training on the health of children, adolescents, and older adults/geriatrics LO 2.3 Within existing training on the physical exam and disease screening; within training on the health of children, adolescents, and older adults/geriatrics, and training on neglected tropical diseases (noma); within maternal and child health
**Module 3: Techniques in oral health promotion and oral disease** Home hygiene practices Social determinants of (oral) health Diet and nutrition Tobacco and alcohol use and cessation (general)	UHC: 1–24 GOH: 2.1.2., 2.1.3., 2.1.6., 3.1.2., 3.2.1., 3.3.3., 3.2.3., 3.3.1.	LO 3.1 Demonstrate proper oral hygiene techniques, including plaque removal and use of fluoridated toothpaste LO 3.2 Provide nutritional advice (meals, snacks, and drinks) and discuss the availability of foods and beverages in the community LO 3.3 Discuss tobacco (or similar) and alcohol use and guide individuals to resources that can support them in reducing use/quitting	LO 3.1 Within primary care training, for example, vaccinations and other preventive services; within training on personal hygiene LO 3.2 Within patient care and primary care training, training on nutrition, and maternal and child health in primary care settings and household settings LO 3.3 Within primary care training, and training on tobacco and alcohol use
**Module 4: School and community-based oral health promotion** School setting oral health campaigns Oral health literacy campaigns and advocacy in community settings Community campaigns to reduce the availability or use of harmful products such as tobacco and alcohol	UHC: 1, 3–7, 9, 10, 12–16, 18, 19, 21–24 GOH: 2.1.3., 2.1.6., 3.1.2., 3.2.1., 3.2.2., 3.2.3., 3.3.1.	LO 4.1 Communicate essential oral health messages for school-aged children in group/classroom settings (e.g. diet, tooth brushing instruction, and oral health education) LO 4.2 Support school personnel in developing oral health advocacy messages in the school setting LO 4.3 Collaborate with local leaders on oral health awareness and literacy campaigns in the community setting (e.g., identify and approach local leadership, develop print media, and hold community events)	LO 4.1 Within training on school-based programs LO 4.2 Within training focused on school-based settings, and trainings on advocacy and health campaigns LO 4.3 Within training focused on community-based settings and health promotion, and trainings on advocacy and health campaigns
**Module 5: Oral health monitoring and data collection at the community level** Monitoring and evaluation process by way of data collection and surveillance, and referral processes in the health-care system CHW’s role within the larger health system	UHC: 1, 3–7, 10, 13–24 GOH: 2.1.1., 2.1.5., 2.2.1., 3.3.2.	LO 5.1 State reasons for collecting and reporting community-level oral health data to the national health system LO 5.2 Perform community oral health data collection for monitoring and surveillance LO 5.3 Outline the country’s oral health system LO 5.4 Demonstrate necessary steps in making a patient referral for further care into the next tier of the system (e.g., community health centers)	LO 5.1 Within training on community-level/school-based data collection and data management/community monitoring training LO 5.2 Within training on community-level/school-based data collection and data management/community monitoring training LO 5.3 Within training on referring patients into the primary care system and on data management for health systems LO 5.4 Within training on referring patients into the primary care system
**Designated Trainer Module and Guide** Building community health workers’ capacity in oral health promotion, and oral disease prevention and control Lesson planning, course delivery, and assessments in a local context and supportive environment	This designated trainer guide consists of five parts: Part 1 discusses their role as the designated trainer. Part 2 describes the course syllabus and provides information about course topics, learning objectives, and potential places for embedding into existing community health workers’ training. Part 3 describes how to assess the competence of their trainees. Part 4 gives advice on course planning and delivery, including creating an enabling environment for delivering this course. Part 5 offers support for monitoring and evaluation in collaboration with chief dental officers or other oral health and health officials. The Summative Assessment Guide, Patient Cases, and Grading Rubrics are included in this module.		

All course materials were written in English and professionally translated into French and Portuguese. These languages were selected by the WHO Regional Office for Africa prior to the start of the project. Because the content is presented in written as well as spoken means, learners may access the content through literary or auditory means. Countries are encouraged to further customize content and language as they see fit. The course design aligns with the OpenWHO platform’s capabilities for delivering information in user-friendly, easily understandable formats in multiple, small, downloadable components. This platform creates maximal flexibility for accessing and storing curriculum content on a variety of devices for field use with or without a stable Internet connection.

#### Evaluation metrics and assessments

Evaluation metrics are an essential component of competency-based training [23]. Evaluation metrics were defined as highly specific practical skills an educator measures in their learners to determine the achievement of each of the learning objectives. Metrics align directly with module content that DTs will use to assess and support learners in achieving competence. The evaluation metrics helped inform the development of the practical course assessments. This assessment approach ensures that the training program is learner-centered, allows for self-guided learning at one’s own pace, and consists of multiple formative and summative assessments. These curricular principles align with competency-based educational norms.

Formative assessments aid in the guided development of the learner by providing periodic feedback. Each module consists of an online formative assessment in the form of a 10-question multiple-choice quiz. Feedback is automatically and immediately generated by the OpenWHO platform for continued learning during the formative assessment. Formative assessments were designed for the application of knowledge and attitudes to case scenarios and patient vignettes rather than simple recitation of facts, aligning with the goals for competency-based education. Learners must pass the formative at 80% or higher, the OpenWHO standard, in order to progress to the next module. Multiple attempts are permitted until the passing score is achieved. Once the learner passes, they can move on to the next module.

Summative assessments serve to assess a learner’s competency at the end of training. A summative assessment was developed in the form of a direct, observational, case-based exercise delivered under the supervision of DTs and was designed to reflect authentic client encounters. The summative assessment guide for evaluators includes implementation guidance for DTs as well as a patient case that can be read or heard by trainees. Then, the learners participate in a face-to-face verbal evaluation with the trainer. The evaluation should be conducted individually (one learner at a time) and should take 15–20 min. Based on the case provided, evaluators will question the learner and listen for key points in their responses. An answer key is provided. It is not required for responses to match the answer key exactly, but rather the evaluator should listen for the key thematic points highlighted in the answer key. To further support the evaluators, key learning points and marking criteria are summarized in a detailed marking rubric. Remediation will be required for any who do not achieve the minimum baseline passing score and will comprise a review of relevant modules where points were missed. After remediation, the summative assessment can be reattempted.

**Figure 3:**
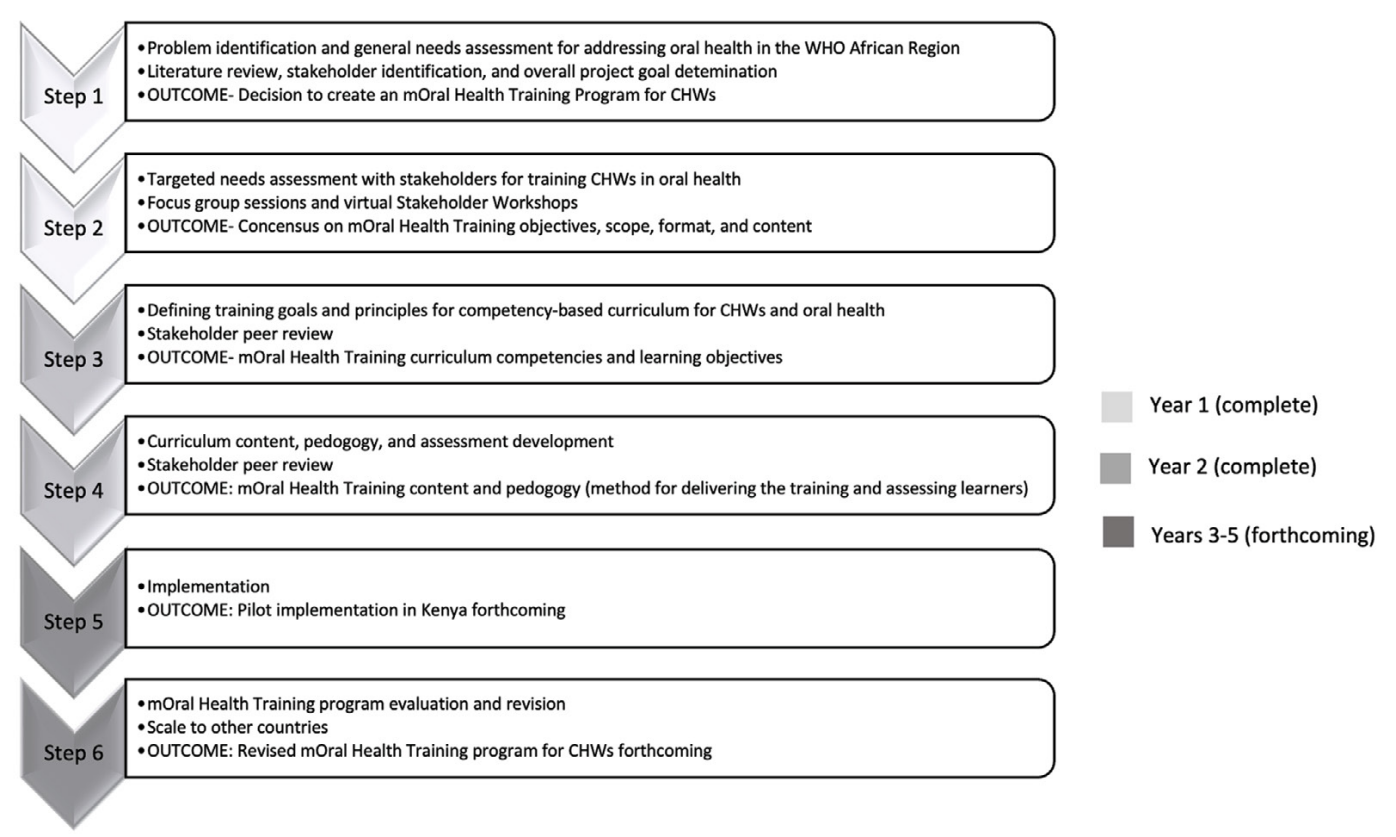
Six-step approach, outcomes, and timeline for curriculum development for the mOral Health Training Program for Community Health Workers in the WHO African Region. CHWs: community health workers; WHO: World Health Organization.

Upon successful completion of the online portion of training, a Record of Achievement is automatically given from OpenWHO, the host of the course. (The course is not administered by the WHO itself). Successful completion of the in-person summative assessment with a DT indicates that a trainee has fully completed the competency-based training on oral health. The in-person summative assessment should be completed before considering the learner successfully trained. A certificate of course completion or other form of acknowledgment may be provided once the summative assessment is passed. Figure 3 outlines the steps, outcomes, and timeline for this project.

## Discussion

This project aligns with global health and workforce development agendas. The Sustainable Development Goals (SDGs) have been instrumental in unifying the world in developing, implementing, and monitoring collaborative strategies for poverty reduction through health improvement. They have strengthened the focus on shared risk factors for NCDs; worked toward UHC; and spurred interprofessional collaboration, partnership, and capacity building for workforce development [26]. The 2016 WHO Global Strategy on Human Resources for Health: Workforce 2030 includes objectives for UHC through equitable, enhanced workforce training and availability responsive to the needs of communities [14]. This mOral Health program was directly informed by, and firmly supports, these global health priorities through building the oral health capacities of primary health-care workers. The 2030 Agenda for Sustainable Development adopted by the United Nations in 2015 recognizes NCDs as a major public health challenge. SDG 3 includes target 3.4 to reduce premature NCD mortality by one‐third by 2030 [7]. The World Health Assembly approved a Resolution on oral health in 2021 at the Seventy-fourth World Health Assembly. The Resolution recommends a shift from the traditional curative approach toward a preventive approach that includes the promotion of oral health within the family, schools, and workplaces, and includes timely, comprehensive and inclusive care within the primary health-care system. The Resolution affirms that oral health should be firmly embedded within the NCD agenda and that oral health-care interventions should be included in national UHC benefit packages, aligning with the SDGs [5].

In response to the mandate outlined in the Resolution, the Secretariat developed the Global strategy on oral health, adopted in May 2022 (decision WHA75.11), and included the Global oral health action plan 2023‒2030 (GOHAP) in the report on NCDs, noted by the Seventy-sixth World Health Assembly in 2023 (WHA76.9). The GOHAP includes a range of actions for Member States, the WHO Secretariat, international partners, civil society organizations, and the private sectors.

CHWs are effective in delivering primary health services to underserved groups’ access to services for the prevention and management of NCDs as part of primary care. Additionally, they are effective in community and social settings for addressing social determinants of health. Guidelines recommend competency-based education that strikes a balance between theory and practice, including a combination of in-person and digital/e-learning modalities with emphasis on local priorities and supervised practical experiences. Supportive training supervision includes observation, assessment, mentorship, and feedback [21]. Our novel oral health curriculum for community-level workers supports each of the above guidelines. Utilization of established, evidence-based guidelines and our strong stakeholder collaboration resulted in content that is adaptable and readily integrated and available under a variety of communication conditions that optimize the chances for program success.

This project is not without limitations. Creating universally adaptable content for such a broad target audience across all regional members of WHO AFRO was challenging, even with such rich input from our lengthy stakeholder feedback processes; it was not feasible to obtain widespread and generalizable input from across the region. In anticipation of future implementation efforts, operationalizing the content via integration into standard operating procedures and scopes of practice expected of community-level providers naturally remains at the discretion of each nation state. Furthermore, barriers may prove to exist regarding the technological support necessary to access the course. Course materials were created to optimize technology and Internet services when available while also allowing for download of content for offline use in rural and remote areas without dependable communication infrastructure in place. Utilization of established, evidence-based guidelines and our strong stakeholder collaboration resulted in content that is adaptable and readily integrated and available under a variety of communication conditions that optimize the chances for program success.

Despite these challenges, this ambitious training program has key strengths. It is, to our knowledge, the first workforce training program directly resulting from strategic objectives and action items from the WHO’s Global Oral Health Action Plan [8]. These include developing innovative workforce models and revising and expanding competency-based education to respond to population oral health needs. Relevant actions also include fostering and exploring innovative oral health workforce models; increasing the capacity of UHC for oral health; and reforming education to prioritize competencies in health promotion, disease prevention, evidence-informed decision-making, digital oral health, service planning, and the social and commercial determinants of health.

Next steps include curriculum implementation, evaluation, and revision, which are Steps and of the six-step approach for curriculum development. Since the launch of the course in July 2023, more than 6000 learners have enrolled in the OpenWHO online course, representing about 10% of the total targeted CHW population across the region. Approximately half of course enrollees have successfully passed all formative assessments and received a Record of Achievement thus far. As part of the next phases of the project, quantitative surveys are being distributed to all enrollees for anonymous, aggregated feedback on the curriculum. Early responses indicate the majority of those enrolled and who have completed the online portion of the course are the intended audience of CHWs. Data are not yet available regarding summative assessments of these learners. The full course with in-person summative assessment by DTs is currently being pilot tested in Kenya, whose Ministry of Health views the launch of this course to fit their national strategic oral health planning goals. Trainee assessments and focus group interviews of CHWs and DTs in Kenya, members from the WHO National Office, and representatives from the Ministry of Health in Kenya will provide crucial feedback. The pilot will assist in identifying barriers and facilitators for successful implementation and offer data-driven approaches for establishing an enabling environment for delivery of the first mOral Health program for CHWs in the region.

## Conclusion

As a result of this project, the WHO AFRO has the first mOral health competency-based CHW training program accessible to all member states. It leverages digital technologies as part of the WHO mHealth initiative and exists in alignment with the WHO Global Strategy on Oral Health. Successful scale of this training program will help to improve the oral health knowledge, skills, and behaviors of CHWs in Africa. Oral health care will become part of the essential package of basic services they provide to their communities. This mOral Health training program lays the groundwork for the development of a tiered health workforce with integrated oral health services that fit into the WHO AFRO agenda for the prevention, control, and management of NCDs across the region.

## Additional Files

The additional files for this article can be found as follows:

Supplementary Appendix 1. World Health Organization Global Competency Framework for Universal Health Coverage.

Supplementary Appendix 2. Global Oral Health Competency Matrix- Competencies deemed highly relevant for Community Health Workers.
