# Supplementary material for: Developing a WHO African Region mOral Health Curriculum for Community Health Workers
Source: Ann Glob Health. 2025 Aug 6;91(1):44. doi: 10.5334/aogh.4655 (PMC12330801; doi:10.5334/aogh.4655)
Supplement: Supplementary Appendix 2. — Global Oral Health Competency Matrix- Competencies deemed highly relevant for Community Health Workers. [file agh-91-1-4655-s2.pdf]

APPENDIX 2.

Global Oral Health Competency Matrix- Competencies deemed highly relevant for Community Health Workers.

1. Knowledge	**1.1.**	**Oral diseases**
	1.1.2.	Understand the essential facts about the etiology of main oral conditions and their symptoms and signs.
	1.1.3.	Describe the impact of oral diseases on well-being and quality of life, as well as its social and economic impact.
	1.1.4.	Identify and assess relevant oral health information and make sound decisions (oral health literacy).
	**1.2.**	**Rist factors and (social) determinants**
	1.2.1.	Identify and describe common risk factors of oral diseases.
	1.2.2.	Identify and describe common (social) determinants of oral diseases.
	1.2.3.	Identify and describe reciprocal links among oral diseases, systemic diseases, and general health.
2. Skills and abilities	**2.1.**	**Disease prevention and health promotion**
	2.1.1.	Conduct an assessment to define oral health needs of the population
	2.1.2.	Understand and apply health promotion and risk reduction strategies (such as healthy eating, cessation of tobacco, and reduction of harmful alcohol use).
	2.1.3.	Promote general oral hygiene knowledge and skills, including toothbrushing twice a day with fluoride toothpaste and cleaning between the teeth
	2.1.4.	Promote and apply other appropriate fluoride interventions.
	2.1.5.	Identify patient populations at increased risk for oral diseases and ensure regular attendance through oral health professionals
	2.1.6.	Promote essential oral health knowledge and skills for expectant mothers and parents to enable appropriate self-care and care for their children.
	**2.2.**	**Disease management**
	2.1.2.	Understand the burden and distribution of oral and associated diseases in specific community and country.
	2.2.2.	Understand and be familiar with the health care system in the community/country.
3. Supporting competencies and principles	**3.1.**	**Interprofessional/intersectoral approach**
	3.1.1.	Demonstrate an interdisciplinary, team-oriented, integrated, and multilevel approach to patient-centered health and oral health care.
	3.1.2.	Recognize the different roles and responsibilities of medical and non-medical professionals in oral health promotion, disease prevention, and, if applicable, treatment, care, and referral.
	3.1.3.	Recognize the areas of specialization in medicine and dentistry.
	**3.2.**	**Cultural and social competence**
	3.2.1.	Demonstrate ethically and culturally competent actions, and show awareness and respect in community settings, customs, differences

		in values, opinions, and practices, cultural norms, and medical cultures (local perceptions of oral health care, attitudes toward dental health, oral care, and seeking professional care)
	3.2.2.	Demonstrate responsive and respectful communication with patients and families, within the oral health team, and with other health professions colleagues
	3.2.3.	Identify, evaluate, and use culturally relevant media and technology.
	**3.3.**	**Professional ethics**
	3.3.1.	Demonstrate professionalism, providing service delivery according to appropriate level of training and ability and representing the profession of dentistry in a responsible manner

Source: Benzian H, Greenspan JS, Barrow J, et al. A competency matrix for global oral health. J Dent Educ 2015;79(4):353-61.
